# An ambispective, observational real-world study of tumor-treating fields for treatment of Chinese patients with newly diagnosed or recurrent/progressive glioblastoma

**DOI:** 10.1093/noajnl/vdag110

**Published:** 2026-04-30

**Authors:** Guanzhang Li, Xiaoguang Qiu, Wei Zhang, Man Hu, Zhiyong Qin, Yang Wang, Jinsong Wu, Wenjie Guo, Xia He, Yongping You, Junxia Zhang, Guanglong Huang, Songtao Qi, Wenbin Ma, Yu Wang, Gang Li, Hua Feng, Fei Li, Yuanyuan Chen, Yonggao Mou, Zhong Wang, Fuyi Liu, Chun Wang, Guangyuan Hu, Kai Shu, Yanhui Liu, Qing Mao, Xiang Wang, Yuan Yang, Juan Dong, Jianmei Hou, Jiayu Fu, Ruijun Xing, Xiang Li, Jun Wan, Hui Zhu, Xuejun Yang, Tao Jiang

**Affiliations:** Beijing Neurosurgical Institute, Capital Medical University, Beijing, China; Beijing Tiantan Hospital, Capital Medical University, Beijing, China; Center of Brain Tumor, Beijing Institute for Brain Disorders, Beijing, China; China National Clinical Research Center for Neurological Diseases, Beijing, China (G.L., X.Q., W.Z., T.J.); Beijing Neurosurgical Institute, Capital Medical University, Beijing, China; Beijing Tiantan Hospital, Capital Medical University, Beijing, China; Center of Brain Tumor, Beijing Institute for Brain Disorders, Beijing, China; China National Clinical Research Center for Neurological Diseases, Beijing, China (G.L., X.Q., W.Z., T.J.); Beijing Neurosurgical Institute, Capital Medical University, Beijing, China; Beijing Tiantan Hospital, Capital Medical University, Beijing, China; Center of Brain Tumor, Beijing Institute for Brain Disorders, Beijing, China; China National Clinical Research Center for Neurological Diseases, Beijing, China (G.L., X.Q., W.Z., T.J.); Department of Radiation Oncology, Cancer Hospital of Shandong First Medical University, Jinan, China; Department of Neurosurgery, Huashan Hospital, Fudan University, Shanghai, China; Department of Radiation Oncology, Huashan Hospital, Fudan University, Shanghai, China; Department of Neurosurgery, Huashan Hospital, Fudan University, Shanghai, China; Department of Radiation Oncology, Jiangsu Cancer Hospital, Nanjing, China; Department of Radiation Oncology, Jiangsu Cancer Hospital, Nanjing, China; Department of Neurosurgery, Jiangsu Provincial Hospital, Nanjing, China; Department of Neurosurgery, Jiangsu Provincial Hospital, Nanjing, China; Department of Neurosurgery, Nanfang Hospital, Southern Medical University, Guangzhou, China (G.H., S.Q.); Department of Neurosurgery, Nanfang Hospital, Southern Medical University, Guangzhou, China (G.H., S.Q.); Department of Neurosurgery, Peking Union Medical College Hospital, Beijing, China; Department of Neurosurgery, Peking Union Medical College Hospital, Beijing, China; Department of Neurosurgery, Qilu Hospital of Shandong University, Jinan, China; Department of Neurosurgery, Southwest Hospital of Army Medical University, Chongqing, China; Department of Neurosurgery, Southwest Hospital of Army Medical University, Chongqing, China; Department of Radiation Oncology, Sun Yat-sen University Cancer Center, Guangzhou, China; Department of Neurosurgery, Sun Yat-sen University Cancer Center, Guangzhou, China; Department of Neurosurgery, The First Affiliated Hospital of Soochow University, Suzhou, China; Department of Neurosurgery, The Second Affiliated Hospital, Zhejiang University School of Medicine, Hangzhou, China; Department of Neurosurgery, The Second Affiliated Hospital, Zhejiang University School of Medicine, Hangzhou, China; Department of Radiation Oncology, Tongji Hospital, Tongji Medical College, Huazhong University of Science and Technology, Wuhan, China; Department of Neurosurgery, Tongji Hospital, Tongji Medical College, Huazhong University of Science and Technology, Wuhan, China; Department of Neurosurgery, West China Hospital, Sichuan University, Chengdu, China; Department of Neurosurgery, West China Hospital, Sichuan University, Chengdu, China; Department of Neurosurgery, West China Hospital, Sichuan University, Chengdu, China; Department of Neurosurgery, West China Hospital, Sichuan University, Chengdu, China; Clinical Development, Oncology, Zai Lab (Shanghai) Co., Ltd, Shanghai, China; Clinical Development, Oncology, Zai Lab (Shanghai) Co., Ltd, Shanghai, China; BioStatistics, Zai Lab (Shanghai) Co., Ltd, Shanghai, China; BioStatistics, Zai Lab (Shanghai) Co., Ltd, Shanghai, China; Medical Affairs, Zai Lab (Shanghai) Co., Ltd, Shanghai, China; Medical Affairs, Zai Lab (Shanghai) Co., Ltd, Shanghai, China; Medical Affairs, Zai Lab (Shanghai) Co., Ltd, Shanghai, China; Beijing Tsinghua Changgung Hospital, Tsinghua University, Beijing, China; Beijing Neurosurgical Institute, Capital Medical University, Beijing, China; Beijing Tiantan Hospital, Capital Medical University, Beijing, China; Center of Brain Tumor, Beijing Institute for Brain Disorders, Beijing, China; China National Clinical Research Center for Neurological Diseases, Beijing, China (G.L., X.Q., W.Z., T.J.)

**Keywords:** China, glioblastoma, real-world study, tumor-treating fields

## Abstract

**Background:**

Glioblastoma is the most common and aggressive central nervous system malignancy with poor prognosis. Tumor-treating fields (TTFields), approved in China in May 2020, represents a significant advancement in GBM treatment. Leveraging the extensive real-world data accumulated in the Chinese Medical Information and Big Data Association (CHMIA) database, this study aims to assess clinical outcomes of Chinese patients with glioblastoma on TTFields therapy.

**Methods:**

This ambispective, observational study assessed post-marketing safety (data cut-off: November 15, 2021) and effectiveness (data cut-off: May 18, 2024) of TTFields in Chinese patients with newly diagnosed glioblastoma (ndGBM) and recurrent glioblastoma (rGBM). Safety outcomes included incidence and severity of skin adverse events (AEs); effectiveness was measured by overall survival (OS) and landmark OS rates.

**Results:**

Of 648 patients screened for this study, 315 were eligible and enrolled for analysis (ndGBM: *n* = 210; rGBM: *n* = 105). In the ndGBM cohort, the median OS was 19.9 months (95% CI: 13.4-24.8), with a 12-month OS rate of 63.5% (95% CI: 54.8-71.0). In the rGBM cohort, the median OS was 8.1 months (95% CI: 5.3-9.8), and 3-month and 6-month OS rates were 80.2% (95% CI: 70.4-87.1) and 58.7% (95% CI: 47.0-68.7), respectively. Most treatment-emergent AEs (TEAEs), including skin TEAEs, were mild to moderate (Grade 1-2).

**Conclusions:**

This study provided the largest real-world dataset to-date on TTFields in Chinese patients with glioblastoma. These patients showed survival outcomes similar to those in prior pivotal studies, without new safety concerns identified, supporting its use in Chinese glioblastoma population.

Key Points

- The largest real-world study of TTFields in Chinese patients with GBM to date.
- ndGBM patients achieved a median OS of 19.9 months, in line with previous studies.
- rGBM patients had a median OS of 8.1 months, at the high end of historical ranges.

Importance of the Study

Tumor-treating fields (TTFields) represent a significant advancement in the treatment of glioblastoma, a highly aggressive central nervous system malignancy with poor prognosis. Leveraging extensive data from the Chinese Medical Information and Big Data Association database, our study provides the largest dataset to date to evaluate the clinical outcomes among Chinese patients with glioblastoma on TTFields therapy. Our findings demonstrated that TTFields therapy yielded median overall survival of approximately 20 months in patients with newly diagnosed GBM and over 8 months in patients with recurrent GBM, aligning with results from pivotal international trials despite potential variations in ethnicity and clinical practice, without new safety signals identified. These findings support the use of TTFields in Chinese clinical practice. This study has validated the efficacy and safety of TTFields therapy in Chinese patients with glioblastoma and will inform future updates to Chinese clinical guidelines for glioblastoma.

Glioblastoma is the most common and aggressive central nervous system (CNS) malignancy, accounting for half of all primary malignant brain tumors.^1^^,^^2^ Glioblastoma has an estimated average incidence of 3.3 per 100,000 person-years, and is more common in people of older age and in males.^1^ With the traditional standard of care (surgical resection, chemoradiotherapy, and maintenance chemotherapy with temozolomide [TMZ]), the median overall survival (OS) is approximately 15 months, with a five-year survival rate of 6.9%, for patients with newly diagnosed glioblastoma (ndGBM).^1^^,^^3^ Nearly all glioblastoma tumors recur after treatment. With no established standard of care that can effectively manage recurrent glioblastoma (rGBM), the median OS is estimated to be only 2-9 months after recurrence.^4^^,^^5^ In China, the overall incidence of brain tumors is 6.2 per 100,000 person-years, with glioblastoma comprising 8.56% of the cases.^2^^,^^6^ Large-scale, systematic epidemiological studies of glioblastoma in China are scarce. A retrospective study conducted in the Glioma Center of Beijing Tiantan Hospital showed a median OS of 14.4 months and a 5-year OS rate of 9% in 254 patients with Grade IV glioblastoma.^7^

TTFields represents a major advancement in the treatment of glioblastoma. TTFields are low-intensity, intermediate frequency (eg, 200 kHz) alternating electric fields delivered via transducer arrays placed on the skin surrounding the tumor site.^8^ With mechanistic complexity yet to be fully unraveled, TTFields is best known for inducing tumor cell death by disrupting mitosis.^8^ The pivotal EF-11 trial, published in 2012, was the first phase 3 randomized controlled trial (RCT) to evaluate TTFields therapy in patients with rGBM.^9^ While no significant difference in OS was observed between the TTFields monotherapy group and the group on physician’s choice chemotherapy (median: 6.6 vs 6.0 months, *P* = .27), TTFields therapy improved patients’ quality of life and showed a more favorable adverse event (AE) profile primarily limited to skin irritation from transducer arrays.^9^ In 2014, the post-marketing registry PRiDe reported a notably longer median OS for rGBM patients treated with TTFields therapy in clinical practice compared to the EF-11 trial (9.6 vs 6.6 months; HR 0.66, 95% CI: 0.50-0.86, *P* = .0003).^10^ Subsequently, the pivotal EF-14 RCT demonstrated significantly improved OS in patients with ndGBM receiving TTFields therapy plus TMZ compared to those receiving TMZ alone (median: 20.9 vs 16.0 months, *P* < .001).^11^

TTFields therapy was approved by the United States Food and Drug Administration for treating rGBM and ndGBM in 2011 and 2015, respectively, constituting the only therapeutic innovation poised for integration into standard of care over the past 2 decades since TMZ’s approval for rGBM in 2009. It has also gained approval in other markets, including the European Union, Switzerland, Japan, and China. In May 2020, TTFields therapy was approved by the National Medical Products Administration (NMPA) in China as concomitant therapy with TMZ for treatment of ndGBM and as monotherapy for treatment of rGBM. The Glioma Diagnosis and Treatment Guideline (2022 edition) in China recommended TTFields therapy as treatment for ndGBM (evidence level 1) and rGBM (evidence level 2).^12^

Being a noninvasive therapeutic modality, TTFields therapy is generally well tolerated, as shown in the 2 pivotal RCTs (including a total of 586 patients with GBM) and in global post-marketing surveillance (including over 25,000 patients with CNS malignancies).^9^^,^^11^^,^^13^ The most common AEs observed with TTFields therapy are localized, manageable, and nonserious skin reactions in patients with glioblastoma and in the patient population with CNS malignancies in general.^9^^,^^11^^,^^13^ However, in China, data remain limited on the real-world effectiveness and safety of TTFields therapy.

This ambispective, observational study aims to describe comprehensively the real-world safety and effectiveness of TTFields therapy in Chinese patients with ndGBM or rGBM by leveraging an established data collection framework, namely, the Chinese Medical Information and Big Data Association (CHMIA). The CHMIA-GBM database is a national-level database dedicated to patients with glioblastoma. It collects a suite of patient information, including demographics, clinical details, and follow-up data, through a patient management platform that integrates data collection with physician-patient interactions. The CHMIA-GBM database thus represents a repository of nationwide data on the real-world management of patients with glioblastoma in China, which this study seeks to fully capitalize on.

## Methods

### Data Source

The CHMIA-GBM database is a national-level database dedicated to patients with glioblastoma in China. To ensure data quality, the database implements a complete data quality management system, encompassing the evaluation standards and metrics for data collection, processing, utilization, and security. A professional data management team and quality control team are responsible for verifying the data to ensure data security and improve data quality. The data undergo structural standardization, data standardization, and model transformation, ultimately presented in a structured and standardized format conducive for statistical analysis. This process ensures data consistency and comparability, providing a reliable foundation for subsequent research. The database mainly consists of 2 parts: (1) patient information (including demographics, relevant medical records, examination images related to hospital visits) and (2) out-of-hospital follow-up data (including follow-up information on patients’ quality of life).

Data in the CHMIA-GBM database are collected through the “Xiao Yi Guan Ai” official account, which is an integrated platform for patient-physician communication, patient follow-up and data collection, on WeChat, a popular social media platform in China. The platform consists of three parts: mobile terminal for physicians, mobile terminal for follow-up personnel, and mobile terminal for patients. It provides a convenient channel for patients and healthcare professionals to keep medical records and follow-up data, including image uploading of medical records by patients and data entry by follow-up personnel for recording patient health file data, thereby pooling data from both inside and outside of the hospital. This not only enables physicians to acquire more complete patient information in real-time for efficient patient management inside and outside the hospital, but also helps generate an ever-growing dataset that can support relevant research work on real-world evidence. All data thus collected through the platform were deidentified and entered into the CHMIA-GBM database.

### Study Design, Participants, and Data Collection

**Figure 1.**
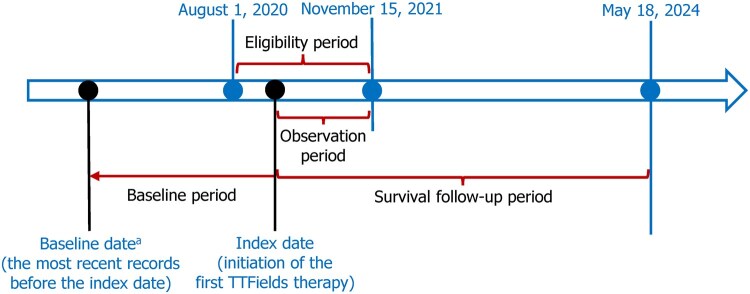
Study design.^a^The baseline date occurred before the index date and might or might not fall within the eligiblity period.

This ambispective, observational real-world study evaluated the post-marketing safety (retrospectively) and effectiveness (retrospectively and prospectively) of TTFields therapy in Chinese patients with glioblastoma. Eligible patients had a histologically confirmed diagnosis of glioblastoma, received TTFields therapy at least once (regardless of the duration) between August 1, 2020 and November 15, 2021 (inclusive), which was defined as the eligibility period (Figure 1), and were aged ≥22 years on the start date of first TTFields therapy (the index date). Patients were excluded if their key baseline data were unavailable, including pathological diagnosis, World Health Organization (WHO) grading, disease status (newly diagnosed or recurrent/progressive), and start date of TTFields therapy. Eligible patients were divided into 2 cohorts: (1) the ndGBM cohort comprising patients with newly diagnosed WHO Grade IV astrocytoma, and (2) the rGBM cohort consisting of patients with recurrent or progressive diseases (by definition, patients should have received surgical resection followed by radiation therapy [if applicable] before the recurrence or progression).

Patients’ eligibility was retrospectively evaluated based on records from the eligibility period. Baseline data were retrospectively retrieved from the CHMIA-GBM database based on the most recent records available before the index date (Figure 1). Safety data during TTFields therapy were retrospectively collected based on CHMIA records from the index date to the end of follow-up within the observation period (data cut-off: November 15, 2021; Figure 1). Survival data were collected both retrospectively from the CHMIA-GBM records during the observation period and prospectively via phone calls conducted by investigators during the survival follow-up period (data cut-off: May 18, 2024; Figure 1). Site contacting, Ethics Committee review and approval, and preparation of documents required by relevant regulations were conducted during the documentation period (October 2022-May 2024).

The study protocol and any amendments thereof were reviewed and approved by the Medical Ethics Committee of Beijing Tiantan Hospital, Capital Medical University. For the retrospective data collection, the data were collected through the “Xiao Yi Guan Ai” official account, where patient users agreed to the privacy terms that permit the use of their information for research purposes. For prospective survival follow-up, informed written consent was obtained from all participating patients. Patient data were stored in accordance with the local regulations for data storage. The study is reported in this manuscript in accordance with the Strengthening the Reporting of Observational Studies in Epidemiology (STROBE) guidelines.

### Treatment Regimen

The User Manual of the TTFields generating device advises patients to use the device for a minimum of 18 hours per day.^14^ TTFields therapy is indicated for use with TMZ for treating ndGBM, and as monotherapy for treating rGBM. The use of additional concomitant anti-tumor treatments was as per investigators’ decisions based on individual patients’ clinical contexts.

### Outcomes

The safety outcomes were the incidence and severity of skin AEs. Treatment-emergent AEs (TEAEs) were defined as AEs occurring within the period from the start of the patient’s first use of TTFields therapy until 30 days after the last TTFields therapy. TEAEs were coded using the Medical Dictionary for Regulatory Activities (MedDRA) Version 26.0. The severity of TEAEs was graded according to the National Cancer Institute-Common Terminology Criteria for Adverse Events (NCI CTCAE) version 5.0.

Effectiveness outcomes included landmark real-world OS rates at specific time points (3-month, 6-month, 9-month, and 12-month when applicable) for the ndGBM cohort, and real-world OS and landmark real-world OS rates at specific time points (3-month and 6-months when applicable) for the rGBM cohort. OS was defined as the duration from the initiation of the first TTFields therapy to the date of death from any cause. The rules for handling missing data on date of death are detailed in Supplementary Methods.

### Statistical Analysis

This study was designed as an ambispective real-world study, including a prospective follow-up component focused on survival data collection, with no prespecified statistical hypotheses. The sample size was determined based on the number of eligible patients identified during the eligibility period. All eligible patients who received any duration of TTFields therapy constituted the safety analysis set (SAS), which was the primary analysis set for both safety and effectiveness analyses. Demographics and baseline characteristics were summarized descriptively. The incidence of AEs was reported as number and percentage of patients. For the analysis of OS, point estimates of median OS and landmark OS rates were calculated using the Kaplan-Meier method; 95% CIs of medians were calculated based on the Brookmeyer-Crowley method and 95% CIs of survival rates were estimated using the Greenwood method. The median follow-up time was calculated using the reverse Kaplan-Meier method. To assess the robustness of the OS results, a sensitivity analysis was performed to evaluate the potential bias to OS by censored patients. The inverse probability of censoring weighting (IPCW) method was adopted to control for the impact of informative censoring on survival estimates, taking into consideration the following prognostic covariates: sex, age category (<65 vs ≥65 years), Karnofsky Performance Score (KPS) category (<90 vs 90-100), isocitrate dehydrogenase 1 (*IDH1*) mutation status, methylguanine-DNA-methyltransferase (*MGMT*) promoter methylation status, 1p/19q co-deletion status, and telomerase reverse transcriptase (*TERT*) promoter mutation status. Subgroup OS analyses were also performed for each cohort by sex (male vs female), age (<65 vs ≥65 years old), baseline KPS (<90 vs 90-100), and *MGMT* promoter methylation status (methylated vs unmethylated).

## Results

### Study Population and Baseline Characteristics

**Figure 2.**
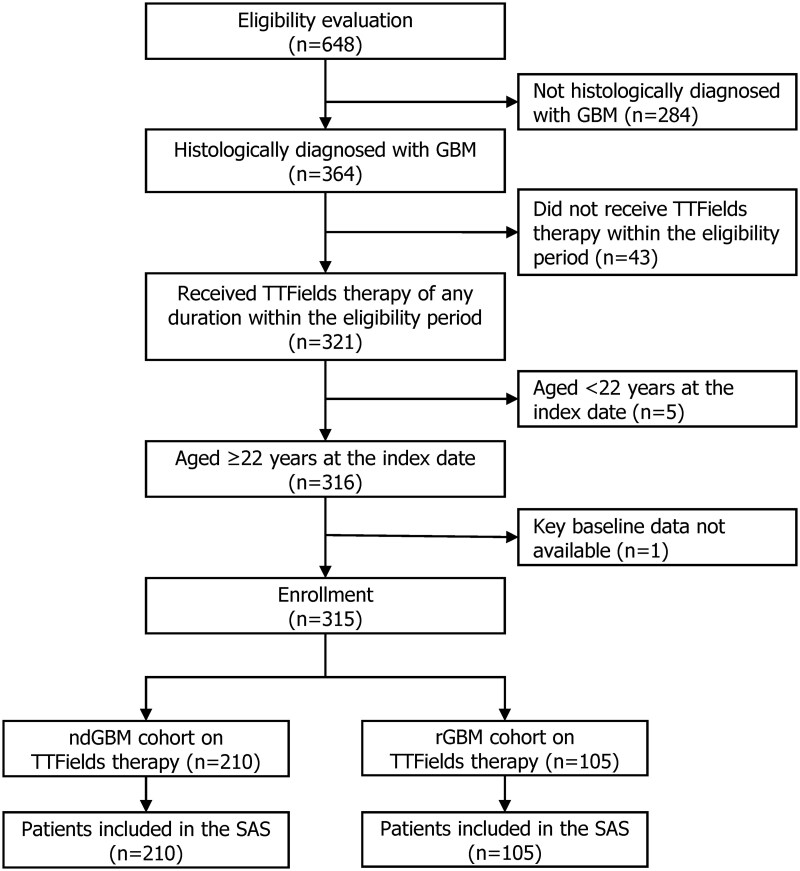
Patient disposition. ndGBM, newly diagnosed glioblastoma; rGBM, recurrent/progressive GBM; SAS, safety analysis set.

A total of 648 patients in the database were screened for this study. Eventually, 333 (51.4%) patients did not meet the eligibility criteria and thus were excluded, and the remaining 315 (48.6%) patients were eligible and constituted the SAS, including 210 patients in the ndGBM cohort and 105 patients in the rGBM cohort (Figure 2).

The median ages in the ndGBM and rGBM cohorts were 53.0 and 54.0 years, respectively, with most aged <65 years (83.3% ndGBM, 83.8% rGBM) and being male (55.7% ndGBM, 58.1% rGBM) (Table 1). In the ndGBM cohort, 48.1% (*n* = 101) of patients had a KPS of <90 and 46.7% (*n* = 98) had a KPS of 90-100. In the rGBM cohort, most patients had a KPS of <90 (71.4%, *n* = 75) and only 21.0% (*n* = 22) had a KPS of 90-100 (Table 1). Most patients had wild-type *IDH1* (82.4% ndGBM, 68.6% rGBM). *MGMT* promoter was methylated in 33.3% of patients in the ndGBM cohort and 41.9% of patients in the rGBM cohort (Table 1). Almost all patients had undergone surgery for the purpose of treating glioblastoma before starting TTFields therapy (ndGBM, 97.1%; rGBM, 94.3%), with gross total resection being the main type (66.7% ndGBM, 56.2% rGBM).

**Table 1.** Baseline demographic and clinical characteristics

Characteristic	ndGBM cohort (*n* = 210)	rGBM cohort (*n* = 105)	Overall (*N* = 315)
Median age (Q1, Q3), years	53.0 (42.0, 63.0)	54.0 (44.0, 58.0)	54.0 (43.0, 62.0)
Age, *n* (%)			
<65 years	175 (83.3)	88 (83.8)	263 (83.5)
≥65 years	35 (16.7)	17 (16.2)	52 (16.5)
Male, *n* (%)	117 (55.7)	61 (58.1)	178 (56.5)
KPS, *n* (%)			
<90	101 (48.1)	75 (71.4)	176 (55.9)
90-100	98 (46.7)	22 (21.0)	120 (38.1)
Missing	11 (5.2)	8 (7.6)	19 (6.0)
Histology, *n* (%)			
Glioblastoma	209 (99.5)	104 (99.0)	313 (99.4)
Other	1 (0.5)	1 (1.0)	2 (0.6)
*IDH1* status, *n* (%)			
Mutated	31 (14.8)	17 (16.2)	48 (15.2)
Wild type	173 (82.4)	72 (68.6)	245 (77.8)
Not tested	3 (1.4)	6 (5.7)	9 (2.9)
Unknown	3 (1.4)	10 (9.5)	13 (4.1)
*MGMT* promoter methylation status, *n* (%)			
Methylated	70 (33.3)	44 (41.9)	114 (36.2)
Unmethylated	72 (34.3)	28 (26.7)	100 (31.7)
Not tested	30 (14.3)	11 (10.5)	41 (13.0)
Unknown	38 (18.1)	22 (21.0)	60 (19.0)
1p/19q co-deletion status, *n* (%)			
Co-deleted	9 (4.3)	4 (3.8)	13 (4.1)
Non-co-deleted	95 (45.2)	33 (31.4)	128 (40.6)
Not tested	50 (23.8)	35 (33.3)	85 (27.0)
Unknown	56 (26.7)	33 (31.4)	89 (28.3)
*TERT* promoter mutation status, *n* (%)			
Mutated	48 (22.9)	17 (16.2)	65 (20.6)
Wild type	29 (13.8)	9 (8.6)	38 (12.1)
Not tested	9 (4.3)	2 (1.9)	11 (3.5)
Unknown	124 (59.0)	77 (73.3)	201 (63.8)
Surgery (including biopsy), *n* (%)	207 (98.6)	101 (96.2)	308 (97.8)
Surgery^a^ for the purpose of treating GBM, *n* (%)	204 (97.1)	99 (94.3)	303 (96.2)
Gross total resection	140 (66.7)	59 (56.2)	199 (63.2)
Partial resection	29 (13.8)	18 (17.1)	47 (14.9)
Missing	35 (16.7)	22 (21.0)	57 (18.1)
Surgical site, *n* (%)			
Supratentorial	174 (82.9)	80 (76.2)	254 (80.6)
Supratentorial + Infratentorial	3 (1.4)	1 (1.0)	4 (1.3)
Infratentorial	2 (1.0)	2 (1.9)	4 (1.3)
Missing	25 (11.9)	16 (15.2)	41 (13.0)
Radiotherapy, *n* (%)	180 (85.7)	65 (61.9)	245 (77.8)
Prior radiotherapy	140 (66.7)	62 (59.0)	202 (64.1)
Concomitant radiotherapy	63 (30.0)	10 (9.5)	73 (23.2)
Pharmacological anti-tumor treatment^a^, *n* (%)	71 (33.8)	28 (26.7)	99 (31.4)
Alkylating agents	70 (33.3)	27 (25.7)	97 (30.8)
VEGF/VEGFR inhibitors	3 (1.4)	4 (3.8)	7 (2.2)
Other TKIs	0 (0.0)	3 (2.9)	3 (1.0)

a Treatment history prior to initiating TTFields therapy. 1p/19q, short arm chromosome 1 and long arm chromosome 19; IDH1, isocitrate dehydrogenase 1; KPS, Karnofsky performance score; MGMT, methylguanine-DNA-methyltransferase; ndGBM, newly diagnosed glioblastoma; rGBM, recurrent/progressive glioblastoma; TERT, telomerase reverse transcriptase; TKI, tyrosine kinase inhibitors; VEFGR, vascular endothelial growth factor receptor; VEGF, vascular endothelial growth factor.

### Concomitant Treatments

While receiving TTFields therapy, 4.8% of patients received at least one surgery for the purpose of treating glioblastoma in both the ndGBM cohort (*n* = 10) and the rGBM cohort (*n* = 5). Pharmacological antitumor treatment was received by 63.3% (*n* = 133) and 48.6% (*n* = 51) of patients in the ndGBM and the rGBM cohorts, respectively; the most commonly used drugs were alkylating agents (other than TMZ) (60.0% [*n* = 126] ndGBM, 39.0% [*n* = 41] rGBM), followed by VEGF/VEGFR inhibitors (8.6% [*n* = 18] ndGBM, 14.3% [*n* = 15] rGBM).

### Overall Survival

**Figure 3.**
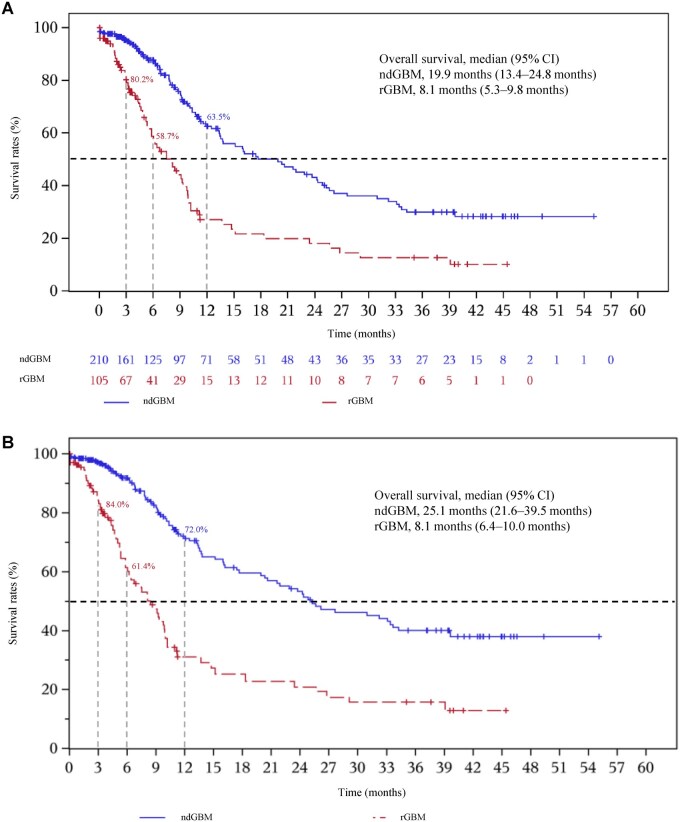
Kaplan-Meier curves for overall survival in (A) the main analysis and (B) the sensitivity analysis by the IPCW method. CI: confidence interval; IPCW: inverse probability of censoring weighting; ndGBM, newly diagnosed glioblastoma; OS, overall survival; rGBM, recurrent/progressive glioblastoma.

As of the data cutoff date of May 18, 2024, in the ndGBM cohort, the median follow-up was 13.2 months (95% CI: 10.9-37.3) over the ambispective survival follow-up period. A total of 87 OS events occurred, and the median OS was 19.9 months (95% CI: 13.4-24.8); the OS rate at 12 months was 63.5% (95% CI: 54.8-71.0) (Figure 3A). In the sensitivity analysis based on the IPCW method, the median OS was 25.1 months (95% CI: 21.6-39.5) and the 12-month OS rate was 72.0% (95% CI: 69.2-74.8) (Figure 3B), which supported the robustness of the main analysis results. Subgroup OS analyses for the ndGBM cohort by age, sex, KPS, and *MGMT* promoter methylation status have been summarized in Supplementary Table S1.

As of the data cutoff date of May 18, 2024, the rGBM cohort had a median follow-up of 35.1 months (95% CI: 10.9-40.0) over the ambispective survival follow-up period. A total of 64 OS events occurred, the median OS was 8.1 months (95% CI: 5.3-9.8), and the 3-month and 6-month OS rates were 80.2% (95% CI: 70.4-87.1) and 58.7% (95% CI: 47.0-68.7), respectively (Figure 3A). The sensitivity analysis based on the IPCW method yielded a median OS of 8.1 months (95% CI: 6.4-10.0) and 3-month and 6-month OS rates of 84.0% (95% CI: 82.4-85.5) and 61.4% (95% CI: 55.5-67.2), respectively (Figure 3B), which supported the robustness of the main analysis. Subgroup OS analyses for the rGBM cohort by age, sex, KPS, and *MGMT* promoter methylation status have been summarized in Supplementary Table S2.

### Safety

As of the end of data collection (excluding prospective survival follow-up), the median follow-up duration was 3.8 months (range: 1.7-7.8) for the overall population. Overall, 52.4% (165/315) of patients experienced any TEAE and 49.5% (156/315) experienced skin TEAEs, and the majority of the TEAEs or skin TEAEs were of Grade 1-2 (Table 2). The TEAEs reported in ≥5% of patients included rash (23.8%), skin ulcer (19.4%), pruritus (14.6%), and erythema (7.9%). A total of 12 patients (3.8%) experienced Grade ≥3 skin TEAEs, including 9 patients from the ndGBM cohort and 3 patients from the rGBM cohort. Grade ≥3 TEAEs reported in ≥2 patients included skin ulcer (1.9%), rash (1.3%), erythema (0.6%), and skin pain (0.6%) (Table 2).

**Table 2.** Summary of TEAEs and Grade ≥3 skin TEAEs (≥2 patients)

Events, *n* (%)	ndGBM cohort (*n* = 210)	rGBM cohort (*n* = 105)	Overall (*N* = 315)
Any TEAE^a^	115 (54.8)	50 (47.6)	165 (52.4)
Any skin TEAE	111 (52.9)	45 (42.9)	156 (49.5)
Any Grade ≥3 skin TEAE**^b^**	9 (4.3)	3 (2.9)	12 (3.8)
Skin ulcer	5 (2.4)	1 (1.0)	6 (1.9)
Rash	3 (1.4)	1 (1.0)	4 (1.3)
Erythema	1 (0.5)	1 (1.0)	2 (0.6)
Skin pain	2 (1.0)	0 (0.0)	2 (0.6)

a If the onset date of an AE was missing, it was regarded as a TEAE based on conservative principles.

b All Grade ≥3 TEAEs were skin TEAEs; Grade 4 skin TEAEs were reported in 3 (1.4%) patients in the ndGBM cohort and no patient in the rGBM cohort; no Grade 5 skin TEAE was reported.

ndGBM: newly diagnosed glioblastoma; rGBM: recurrent/progressive glioblastoma; TEAE: treatment-emergent adverse event.

## Discussion

This study is the first to leverage the resource of a glioblastoma-dedicated database after TTFields therapy’s approval in China, and provides real-world data that fill the current evidence gap on the post-marketing safety and effectiveness of TTFields therapy in Chinese patients with glioblastoma. With a study population representative of Chinese patients with glioblastoma in routine clinical practice, this study demonstrated the effectiveness of TTFields therapy consistent with key results in the literature for both ndGBM and rGBM, with the robustness of the results demonstrated by sensitivity analysis. The incidence of AEs was similar to those reported in previous publications, with no new safety signals detected, indicating TTFields therapy’s manageable safety profile.

For patients with ndGBM receiving TTFields therapy, the median OS in this study (19.9 months) is in line with that in the pivotal RCT of EF-14 (20.9 months)^11^ and the pooled median OS (22.6 months) by meta-analysis of 6 real-world studies from the United States, China, and Czech Republic.^15^ Previous single-center, retrospective studies from several reputable hospitals in China reported median OS of 19.7-24.8 months for patients with ndGBM treated with TTFields therapy (samples sizes ranged: 13-81), which is also generally consistent with the result from the current study.^16–19^ According to hazard ratio analyses from EF-14, the aforementioned meta-analysis, and a Chinese retrospective study (*n* = 63 with TTFields therapy, *n* = 204 without TTFields therapy), compared to no TTFields therapy, receiving TTFields therapy was observed to reduce the risk of death for patients with ndGBM by approximately 40%-60% during in-study follow-up periods.^11^^,^^15^^,^^19^ The numerical consistency in median OS between the current study and the existing body of evidence further supports the notion that the use of TTFields therapy may confer a meaningful real-world survival benefit for patients with ndGBM.

The rGBM cohort of this study showed a median OS of 8.1 months, a value at the higher end of the historically reported range of 2-9 months,^4^^,^^5^ suggesting treatment with TTFields may result in similar survival outcomes as other standard of care therapies for patients with rGBM, without the associated systemic toxicity. This median OS is similar to the 7.4 months reported in a prospective study of patients from the PRiDe registry (registry for all patients in the US treated with TTFields therapy commercially) during 2016-2017.^20^ A *post-hoc* analysis of the EF-11 trial reported a similar median OS of 7.7 months in patients receiving ≥1 course of TTFields therapy, which was longer than that in the chemotherapy control arm.^21^ A *post-hoc* analysis of EF-14 among patients upon first recurrence reported survival benefit with the addition of TTFields therapy versus second-line chemotherapy alone (median OS 11.8 vs 9.2 months, HR = 0.70, *P* = .049).^22^ The trend of real-world survival of the rGBM cohort in the current study is consistent with the survival benefit conferred by TTFields therapy in those earlier studies.

The safety profile of TTFields therapy in this study is consistent with that established in previous clinical and real-world studies, with no new safety signal detected. Most TEAEs were skin AEs, which occurred in 49.5% of patients. This is in line with the global post-marketing safety surveillance data of TTFields therapy from 25,898 patients, where beneath-array skin reactions were the most common treatment-related AE (43%).^13^ In the overall population in this study, most skin AEs were of Grade 1-2, which was also consistent with previous studies, where AEs with TTFields therapy were generally of low grade, nonserious, and mild-to-moderate in severity.^13^^,^^16^^,^^20^ To prevent and better manage skin AEs, it is recommended to perform skin care and treatment with appropriate therapeutic modalities such as skin barrier films, moisturizers, topical corticosteroids, antiperspirants, and antibiotics. When reapplying, shifting array position also helps to prevent or minimize skin AEs.^23^

This study had several limitations. First, the absence of comparator groups limited the study’s ability to assess the benefit of adding on TTFields therapy to the treatment in patients with ndGBM and its standalone or add-on benefit in patients with rGBM. Nevertheless, numerical comparisons with historical references support TTFields therapy as a useful addition to the treatment repertoire to improve the survival of patients with glioblastoma. Second, there were limitations inherent to the retrospective nature of information collection, such as missing data for baseline prognostic biomarker status. Third, specific data on device usage patterns (eg, duration of treatment, device usage rate [ie, percentage of time the device was on]) were not analyzed in this study, which may limit the interpretation of treatment outcomes, as persistence of and adherence to TTFields therapy has been previously shown to influence OS.^15^^,^^16^ For future research, it would be of interest to delineate the real-world usage patterns of TTFields therapy and its influence on effectiveness among Chinese patients with glioblastoma, for example, by analyzing the outcomes in patients with ≥30-day usage and/or different levels of device usage rates. Finally, only a small proportion (16.5%, 52/315) of elderly (≥65-year-old) patients were included. In real-word settings, elderly patients are more likely to face economic barriers to TTFields therapy, as it is covered by private medical insurance only, which is not the primary source of elderly coverage.

This ambispective, observational study provides the largest dataset to date on the real-world clinical outcomes of TTFields therapy in Chinese patients with glioblastoma. The survival outcomes of both patients with ndGBM and rGBM were consistent with results of the pivotal studies, EF-14 and EF-11. The safety results in this study are consistent with the known safety profile of TTFields therapy established through pivotal trials and substantial global post-marketing surveillance, with no new safety signal detected. These findings support the use of TTFields therapy in patients with glioblastoma in China.

## Supplementary Material

vdag110_Supplementary_Data

## Data Availability

The data supporting the findings of this study are available from the corresponding author, Tao Jiang, upon reasonable request.
