# Supplementary material for: An ambispective, observational real-world study of tumor-treating fields for treatment of Chinese patients with newly diagnosed or recurrent/progressive glioblastoma
Source: Neurooncol Adv. 2026 Apr 30;8(1):vdag110. doi: 10.1093/noajnl/vdag110 (PMC13215093; doi:10.1093/noajnl/vdag110)
Supplement: vdag110_Supplementary_Data [file vdag110_supplementary_data.docx]

**SUPPLEMENTARY APPENDIX**

**Supplementary Methods. Handling of Missing Values**

For patients with incomplete dates of death, the rules of imputation were as follows: 1) if the year, month and day of death were all missing, the date of death was imputed as the last date the patient was known to be alive + 1 day; 2) if the month and day of death were missing, the date of death was imputed as January 1 of the recorded year of death (if greater than the last year the patient was known to be alive) or as the last date the patient was known to be alive + 1 day (if recorded year of death equaled the last year known to be alive); 3) if only the day of death was missing, the date of death was imputed as the first day of the recorded month of death (if the year and month greater than the last year and month known to be alive) or as the last date the patient was known to be alive + 1 day (if the recorded year and month of death equaled the last year and month known to be alive). For patients who were lost to follow-up or still alive at the end of the Survival Follow-up Period, the OS was censored at the last date the patient was known to be alive.

**Supplementary Table 1. Subgroup analyses of overall survival in the ndGBM cohort**

| **Subgroup** | **events/N^a^** | **Median OS**  **(95% CI), months** | **OS rate (95% CI), %** | | | |
| --- | --- | --- | --- | --- | --- | --- |
|  |  |  | **3-month** | **6-month** | **9-month** | **12-month** |
| Age |  |  |  |  |  |  |
| <65 years | 68/175 | 20.6  (13.7–27.6) | 95.0  (90.3–97.5) | 87.5  (80.8–91.9) | 77.6  (69.2–84.0) | 64.7  (55.0–72.7) |
| ≥65 years | 19/35 | 13.3  (6.9–24.2) | 96.6  (77.9–99.5) | 88.7  (68.8–96.3) | 67.4  (45.0–82.3) | 58.4  (36.2–75.2) |
| Sex |  |  |  |  |  |  |
| Male | 48/117 | 17.4  (12.5–24.3) | 94.3  (87.7–97.4) | 87.5  (78.9–92.7) | 73.6  (62.5–81.9) | 64.3  (52.2–74.0) |
| Female | 39/93 | 20.6  (11.2–33.4) | 96.6  (89.8-98.9) | 87.9  (77.9–93.6) | 78.4  (66.5–86.4) | 62.7  (49.4–73.4) |
| KPS |  |  |  |  |  |  |
| <90 | 52/101 | 13.3  (9.7–17.7) | 95.7  (88.8–98.4) | 83.6  (73.3–90.2) | 66.9  (54.5–76.6) | 51.5  (38.7–62.9) |
| 90–100 | 32/98 | 25.6  (17.4–NE) | 96.6  (89.8–98.9) | 92.5  (84.1–96.6) | 84.8  (74.1–91.3) | 74.7  (62.3–83.6) |
| *MGMT* promoter methylation status (*post-hoc*) | | | | | | |
| Methylated | 25/70 | 33.4 (20.6-NE) | 97.0 (88.4-99.2) | 91.5 (80.7-96.4) | 83.1 (69.7-90.9) | 76.3 (61.7-85.9) |
| Unmethylated | 40/72 | 11.2  (8.3-16.2) | 95.5 (86.6-98.5) | 82.9  (70.5-90.5) | 61.7 (46.8-73.5) | 45.6 (31.1-59.1) |
| ^a^N is the sample size of the subgroup.  CI, confidence interval; KPS, Karnofsky performance score; ndGBM, newly diagnosed glioblastoma; NE, not estimable; OS, overall survival. | | | | | | |

**Supplementary Table 2. Subgroup analyses of overall survival in the rGBM cohort**

| **Subgroup** | **events/N^a^** | **Median OS**  **(95% CI), months** | **OS rate (95% CI), %** | |
| --- | --- | --- | --- | --- |
|  |  |  | **3-month** | **6-month** |
| Age |  |  |  |  |
| <65 years | 53/88 | 7.5  (5.8–9.9) | 80.3  (69.3–87.6) | 59.6  (46.8–70.3) |
| ≥65 years | 11/17 | 8.1  (1.7–10.0) | 80.0  (50.0–93.1) | 55.0  (25.0–77.3) |
| Sex |  |  |  |  |
| Male | 38/61 | 8.4  (5.3–9.9) | 80.1  (66.9–88.4) | 64.6  (49.5–76.2) |
| Female | 26/44 | 5.8  (4.7–10.0) | 80.8  (63.7–90.4) | 49.6  (30.9–65.9) |
| KPS |  |  |  |  |
| <90 | 47/75 | 6.2  (4.7–9.3) | 79.7  (67.5–87.7) | 52.2  (38.0–64.6) |
| 90–100 | 12/22 | 8.9  (5.3–NE) | 79.6  (54.3–91.8) | 73.5  (47.2–88.1) |
| *MGMT* promoter methylation status (*post-hoc*) | | | | |
| Methylated | 27/44 | 9.9  (6.7-14.8) | 92.8  (79.3-97.6) | 76.2  (59.1-86.9) |
| Unmethylated | 19/28 | 4.5  (2.9-5.3) | 63.7  (40.1-80.0) | 17.6 (4.5-38.0) |
| ^a^N is the sample size of the subgroup.  CI, confidence interval; KPS, Karnofsky performance score; NE, not estimable; OS, overall survival; rGBM, recurrent/progressive glioblastoma. | | | | |
